# Why Is the Correlation between Gene Importance and Gene Evolutionary Rate So Weak?

**DOI:** 10.1371/journal.pgen.1000329

**Published:** 2009-01-09

**Authors:** Zhi Wang, Jianzhi Zhang

**Affiliations:** Department of Ecology and Evolutionary Biology, University of Michigan, Ann Arbor, Michigan, United States of America; Fred Hutchinson Cancer Research Center, United States of America

## Abstract

One of the few commonly believed principles of molecular evolution is that functionally more important genes (or DNA sequences) evolve more slowly than less important ones. This principle is widely used by molecular biologists in daily practice. However, recent genomic analysis of a diverse array of organisms found only weak, negative correlations between the evolutionary rate of a gene and its functional importance, typically measured under a single benign lab condition. A frequently suggested cause of the above finding is that gene importance determined in the lab differs from that in an organism's natural environment. Here, we test this hypothesis in yeast using gene importance values experimentally determined in 418 lab conditions or computationally predicted for 10,000 nutritional conditions. In no single condition or combination of conditions did we find a much stronger negative correlation, which is explainable by our subsequent finding that always-essential (enzyme) genes do not evolve significantly more slowly than sometimes-essential or always-nonessential ones. Furthermore, we verified that functional density, approximated by the fraction of amino acid sites within protein domains, is uncorrelated with gene importance. Thus, neither the lab-nature mismatch nor a potentially biased among-gene distribution of functional density explains the observed weakness of the correlation between gene importance and evolutionary rate. We conclude that the weakness is factual, rather than artifactual. In addition to being weakened by population genetic reasons, the correlation is likely to have been further weakened by the presence of multiple nontrivial rate determinants that are independent from gene importance. These findings notwithstanding, we show that the principle of slower evolution of more important genes does have some predictive power when genes with vastly different evolutionary rates are compared, explaining why the principle can be practically useful despite the weakness of the correlation.

## Author Summary

The fact that functionally more important genes or DNA sequences evolve more slowly than less important ones is commonly believed and frequently used by molecular biologists. However, previous genome-wide studies of a diverse array of organisms found only weak, negative correlations between the importance of a gene and its evolutionary rate. We show, here, that the weakness of the correlation is not because gene importance measured in lab conditions deviates from that in an organism's natural environments. Neither is it due to a potentially biased among-gene distribution of functional density. We suggest that the weakness of the correlation is factual, rather than artifactual. These findings notwithstanding, we show that the principle of slower evolution of more important genes does have some predictive power when genes with vastly different evolutionary rates are compared, explaining why the principle can be practically useful for tasks such as identifying functional non-coding sequences despite the weakness of the correlation.

## Introduction

When referring to any DNA sequence, a popular textbook of cell and molecular biology [1] states that “if it's conserved, it must be important” and calls this “one of the foremost principles of molecular evolution” (p. 416). Here, the word “conserved” means that the sequence has a low rate of evolution such that its orthologs from distantly related species are detectable and alignable. The word “important” means that the sequence has relevance to the wellbeing and fitness of the organism bearing the sequence. The above principle is often used in a comparative context, asserting that functionally more important DNA sequences evolve more slowly. Despite the fact that thousands of biologists accept this principle and use it daily in identifying functionally important DNA sequences, its validity had not been systematically examined until a few years ago when gene importance could be measured at the genomic scale [2]–[10]. Unexpectedly, however, genomic studies of bacteria, fungi, and mammals showed that although the evolutionary rate of a gene is significantly negatively correlated with its importance, the latter only explains a few percent of the total variance of the former [3],[4],[10],[11]. The striking contrast between the wide acceptance and apparent utility of the principle and the weakness of the correlation revealed from genomic analysis of a diverse array of organisms is perplexing.

The perceived theoretical basis of this simple principle is the neutral theory of molecular evolution, which asserts that most nucleotide substitutions during the evolution of a gene are due to random fixations of neutral mutations [12]–[14]. Based on this theory, Kimura and Ohta first predicted that functionally more important genes should evolve slower than less important ones because the former have a lower rate of neutral mutation than the latter [15], although their use of “functional importance” appears to mean “functional constraint on the gene” rather than “importance to the fitness of the organism”. A few years later, Wilson *et al.* separated the two meanings and decomposed the substitution rate of a gene (*k*) into two factors: the probability (*P*) that a random mutation will be compatible with the function of the gene and the probability (*Q*) that an organism can survive and reproduce normally without the gene (i.e., gene dispensability) [16]. Under the simple assumption that a mutation either completely abolishes the function of a gene (with a probability of α = 1−*P*) or does not affect it at all (with a probability of 1−α), we can write the substitution rate of a gene as the sum of the rate of fixation of neutral mutations and that of null mutations. Here, α can also be interpreted as functional density, the effective fraction of sites in a gene (or protein) that are required for its function. Let *u* be the total mutation rate, β = 1−*Q* be the probability that an organism cannot survive or reproduce without the gene (i.e., gene importance or the coefficient of selection against null mutations), *N* be the organism's population size, and *N_e_* be the effective population size. For diploid organisms, we have
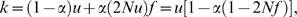
(1)where 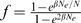 is the probability of fixation of a new null mutation with fitness 0<*Q*<1, under genic selection (i.e, the selection against the null allele is β in homozygotes and β/2 in heterozygotes) [12]. Because *f*<1/(2*N*), *k* is a monotonically decreasing function of α. It is obvious that *k* is also a monotonically decreasing function of β, because the stronger the selection against null mutations, the lower *f* and *k* are. However, note that the above formula also indicates that in large populations, *f* and hence *k* should be relatively insensitive to β except when β is extremely small (i.e., on the order of 1/*N*_e_). In other words, under the simplistic model assumed here, a strong negative correlation between gene importance and evolutionary rate is not expected [6] (see also Text S1 and Figure S1). However, under a more realistic model with the presence of slightly and moderately deleterious mutations, a much stronger correlation between gene importance and evolutionary rate becomes theoretically possible [7]. The strength of the correlation depends on the distribution of the deleterious functional effects of random mutations (Text S1 and Figure S1). Because the true distribution is currently unknown, theories cannot predict precisely the strength of the correlation between gene importance and evolutionary rate. These considerations notwithstanding, the apparent utility of the principle in daily practice and its lack of empirical support from genomewide studies require an explanation.

There are two simple, yet untested, hypotheses that potentially explain the weakness of the observed correlation between gene importance and evolutionary rate. First, the importance of a gene to an organism is now commonly measured by the fitness reduction caused by the deletion of the gene from the genome in a benign lab condition; deleting an important gene reduces the fitness of the organism more than deleting a less important one. But, because lab conditions differ significantly from the natural environments of organisms, gene importance determined in lab may be quite different from that in nature [6],[17]. For example, in rich media, ∼80% of yeast genes are not essential for growth [18]. However, metabolic network analysis and experimental studies showed that most of these dispensable genes are important for growth under other conditions [18],[19], some of which may resemble the natural environments of the species better than rich media. Hence, it is plausible that the weakness of the correlation between gene importance and evolutionary rate is due to inaccuracy in measuring genes' natural importance, which we refer to as the lab-nature mismatch hypothesis. But, measuring gene importance in a species' natural environment is difficult because many species such as the yeast *Saccharomyces cerevisiae* are found in diverse environments that are poorly characterized [20]. Moreover, even if we know the present-day natural environments of a species, they may not reflect the environments where the species lived in the past. These historical environments are crucial because the gene evolutionary rate that is being correlated to gene importance is determined by comparison between species. Nonetheless, if gene importance is measured in many different conditions, we can examine whether the correlation between gene importance and evolutionary rate is much stronger in some conditions than in the benign lab condition, which could at least demonstrate the plausibility of the lab-nature mismatch hypothesis. Here we test this hypothesis in yeast using gene importance measures from both experimental data and computational predictions. The experimental data came from a set of recently published fitness measurements of yeast single-gene-deletion strains under 418 lab stress conditions [19]. We complemented this dataset with *in silico* predictions of importance for metabolic enzyme genes under 10^4^ nutritional conditions, achieved by flux balance analysis (FBA) of reconstructed metabolic networks [21],[22].

Another potential factor influencing the correlation between gene importance (β) and evolutionary rate (*k*) is functional density (α) in Equation 1. If α and β are negatively correlated (i.e., more important genes have lower functional density), the correlation between *k* and β will be weakened. Although there is no reason to believe that α and β are negatively correlated, it is worth verifying using actual data. For a given protein, α may be approximately measured by the fraction of sites in functional domains, which can be computationally predicted.

In this work, we show that neither of the above two hypotheses is correct in yeast. Rather, the weakness of the correlation between gene importance and evolutionary rate is likely to be factual rather than artifactual. We show, however, that the principle of slower evolution of more important genes does have some predictive power when genes with vastly different evolutionary rates are compared, explaining why the principle can be practically useful despite the weakness of the correlation.

## Results/Discussion

### Testing the Lab-Nature Mismatch Hypothesis with Experimental Measures of Gene Importance

The most frequently used yeast gene importance data came from the measures of relative growth rates of 5936 single-gene-deletion yeast strains in the nutritionally rich YPD medium [23]. Recently, the same type of measure was taken for all YPD-viable single-gene-deletion yeast strains under 418 diverse laboratory conditions, of which ∼75% are chemical drug treatments and the rest are environmental stress conditions such as different pHs and temperatures [19]. These two datasets of gene importance are used in our analysis.

Evolutionary rates of *S. cerevisiae* genes are estimated by comparing these genes to their orthologs in related species. Because the functional importance of a gene may change during evolution [10],[24], it is better to use a closely related species for rate estimation. However, when the species are too close, the number of nucleotide substitutions per gene may be insufficient for precise estimation of evolutionary rates. A previous study found the strongest correlation between gene importance and evolutionary rate when *S. cerevisiae* is compared with *S. bayanus* [10]. We thus use this species pair and obtain 3999 genes with identifiable orthologs. Our results remain qualitatively unchanged when several other yeast species were compared with *S. cerevisiae* (data not shown). We use the number of nonsynonymous substitutions per nonsynonymous site (*d*_N_) between orthologs to measure the rate of gene evolution (*k* in Equation 1). Because the mutation rate (*u* in Equation 1) may vary among genes, we also use the ratio between *d*_N_ and the number of synonymous substitutions per synonymous site (*d*_S_) as a measure of *k*/*u* in Equation 1.

**Figure 1:**
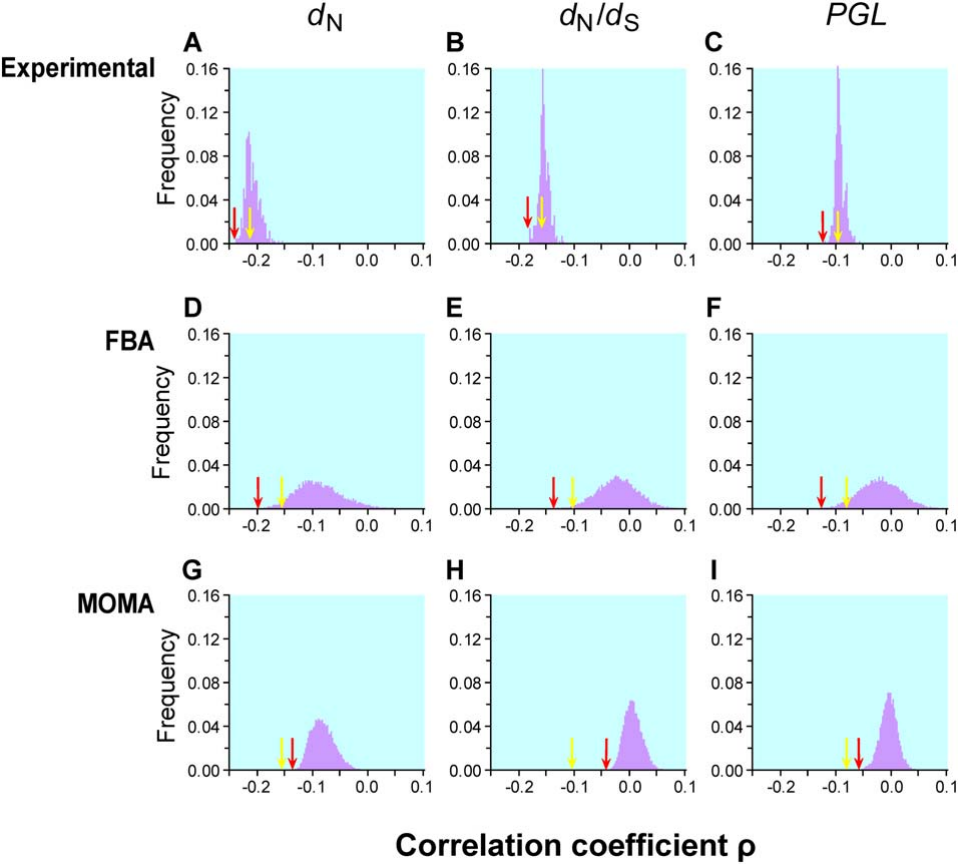
Frequency distributions of Spearman's rank correlation coefficient ρ between gene importance (i.e., fitness reduction upon gene deletion) and evolutionary rate across many conditions. Gene importance is measured by experiments in 418 lab conditions (panels A–C), predicted by FBA for enzyme genes in 10^4^ simulated nutritional conditions (D–F), or predicted by MOMA for enzyme genes in the same 10^4^ conditions (G–I). Gene evolutionary rate is measured by nonsynonymous substitution rate *d*_N_ (A, D, G), nonsynonymous/synonymous rate ratio *d*_N_/*d*_S_ (B, E, H), or propensity for gene loss *PGL* (C, F, I). The yellow arrow in each panel indicates the observed correlation using gene importance values experimentally determined in the YPD medium and the red arrow indicates the strongest correlation across the conditions examined. The numbers of genes used are 3999 for panels A–C, 478 for panels D, E, G, and H, and 546 for panels F and I. The gene number is lower than 546 for panels D, E, G, and H, because some *S. cerevisiae* genes do not have orthologs in *S. bayanus*. The yellow arrow is on the left-hand side of the red arrow in panels G, H, and I, because, under all simulated conditions, MOMA-predicted fitness values have weaker correlations with the evolutionary rates than that observed under YPD.

When gene importance is measured under the nutritionally rich YPD medium, the Spearman's rank correlation coefficient between gene importance (i.e., amount of fitness reduction caused by gene deletion) and *d*_N_ is ρ = −0.2189 (*P*<10^−43^; Figure 1A). Our examination of 418 other lab conditions found the strongest correlation to be ρ = −0.2379 (*P*<10^−51^; Figure 1A). Thus, none of the 418 conditions provides a substantially stronger correlation than what is observed with YPD. Similar results were obtained for the correlation between gene importance and *d*_N_/*d*_S_ (Figure 1B).

Krylov et al. suggested another measure of gene evolutionary rate known as the propensity for gene loss (*PGL*), which is the number of times that a gene is lost during the evolution of a group of species [11]. Although *PGL* and *d*_N_ are correlated with each other [11], they measure the rate of gene evolution from different angles. The correlation between *PGL* and gene importance is expected to be weaker than that between *d*_N_ and gene importance, because mutations that impair gene function only slightly do not matter to gene loss. We estimated *PGL* for each *S. cerevisiae* gene by counting the number of gene loss events on the known phylogeny of 12 fungal species (see Materials and Methods). Consistent with our expectation, the correlation between gene importance and *PGL* is weaker than that between gene importance and *d*_N_ (or *d*_N_/*d*_S_) for both YPD and the other 418 lab conditions (Figure 1C). Regardless, the examination of the 418 lab conditions does not substantially improve the strength of the correlation between gene importance and *PGL*.

### Testing the Lab-Nature Mismatch Hypothesis with Computationally Predicted Gene Importance Values

Because the 418 experimentally examined conditions contain mostly artificial chemical treatments and hence may not cover the diverse natural environments of the yeast, we decide to complement the experimental data with computationally predicted gene importance values for 546 metabolic enzyme genes under 10^4^ conditions generated by random combinations of different nutrients following a sampling strategy that mimics the potential nutritional environments of the wild yeast (see Materials and Methods). We then used two different experimentally validated computational methods to predict the fitness reduction caused by the deletion of each enzyme gene. These methods rely on the reconstructed high-quality yeast metabolic network [25], which contains 632 biochemical reactions associated with 546 enzyme genes after the removal of dead-end reactions [26]. The first method we used is flux balance analysis (FBA). Under the assumption of steady state of every cellular metabolite, FBA maximizes the rate of biomass production under the stoichiometric constraints of all metabolic reactions [22]. Simulation of different nutritional conditions is achieved by setting the boundaries of uptake reaction fluxes and simulation of gene deletion is achieved by constraining the flux of corresponding enzymatic reaction to zero (see Materials and Methods). In our analysis, we consider the FBA-optimized rate of biomass production as the wild-type Darwinian fitness of the cell under the condition specified. The relative fitness of a cell lacking a gene is the FBA-optimized rate of biomass production of the cell, divided by that of the wild-type cell. Previous studies demonstrated that FBA makes excellent qualitative predictions of yeast gene essentiality under typical experimental conditions [18],[25]. A recent study further showed consistent performances of FBA across many different conditions [27]. Following a previous study [28], we approximated the YPD condition in the FBA model and predicted the fitness values of single-gene-deletion yeast strains. We found that the FBA-predicted fitness values correlate well with the experimentally determined fitness values under YPD (Pearson's *r* = 0.562, *P*<10^−41^). We were not able to verify FBA for the other 418 lab conditions because these conditions are difficult to specify in FBA.

Our extensive analysis of 10^4^ simulated conditions identified the strongest correlation between FBA-predicted gene importance and *d*_N_ to be ρ = −0.2186 (*P* = 10^−6^; Figure 1D) for 546 enzyme genes. Although this correlation is 34% stronger than that estimated using experimentally determined gene importance under YPD (ρ = −0.1636, *P* = 6×10^−4^; Figure 1D) for the same set of genes, the fraction of variance in *d*_N_ that is explainable by gene importance is still as low as (−0.2186)^2^ = 4.8%. Similar results are obtained when either *d*_N_/*d*_S_ (Figure 1E) or *PGL* (Figure 1F) is used as a measure of gene evolutionary rate. One interesting observation is that the standard deviation of ρ from the 10^4^ simulated conditions (0.042, 0.037, and 0.037 in Figure 1D, E, and F, respectively) is much greater than that for the 418 experimental conditions (0.013, 0.009, and 0.008 in Figure 1A, B, and C, respectively). Part of this difference is due to the use of essentially all genes in Figure 1A–C but only enzyme genes in Figure 1D–F. However, even when only enzyme genes are considered, the standard deviation of ρ is still smaller for lab conditions (*d*_N_: 0.024; *d*_N_/*d*_S_: 0.021; *PGL*: 0.020) than for the 10^4^ simulated conditions, suggesting that the simulated conditions represent a more diverse set of conditions than the experimental conditions.

FBA assumes that a cell can readjust its metabolic fluxes to achieve the highest possible biomass production immediately after the deletion of any gene, which is probably unrealistic. Segre and colleagues proposed a modified method known as the minimization of metabolic adjustment (MOMA) [29]. Instead of maximizing biomass production upon gene deletion, MOMA minimizes the changes of fluxes from those of the wild-type cell. Empirical examples suggested that MOMA outperforms FBA in predicting gene essentiality and metabolic fluxes [29]. We found that MOMA-predicted fitness values of single-gene-deletion strains are slightly better than FBA-predicted values in correlating with the experimentally determined fitness values in YPD (Pearson's *r* = 0.571, *P*<10^−43^). However, none of the 10^4^ simulated conditions provide a better correlation between MOMA-predicted gene importance and evolutionary rate than the correlation found using experimentally measured gene importance in YPD (Figure 1G–H).

Although we examined 10^4^ simulated conditions, it is possible that they still do not cover the natural conditions of yeast. We simulated 10^5^ additional conditions and found that the distribution of the correlation coefficient ρ (Figure S2) is virtually identical with that from the initial 10^4^ conditions. Because the distribution of ρ is approximately normal, statistically speaking, it is extremely unlikely to obtain a much stronger correlation by examining even 10^6^ conditions. Due to the large amount of computational time required for examining large numbers of conditions and the similarity of the results from 10^4^ and 10^5^ conditions, we used the gene importance values predicted from the 10^4^ conditions in subsequent analysis.

### Testing the Lab-Nature Mismatch Hypothesis using Combinations of Individual Conditions

Because under no single condition, either experimentally examined or computationally simulated, did we find a strong correlation between gene importance and evolutionary rate, and because yeast may have had experienced diverse natural conditions during its evolution, we ask whether we can find combinations of single conditions for which the correlation between gene importance and evolutionary rate is much stronger than that under any single condition. We consider a simple scenario in which gene importance values under different conditions are weighted and linearly combined to form an average gene importance value across all the conditions considered. These weighting coefficients potentially represent the (unknown) relative durations of the conditions where the yeast has lived. We identify these coefficients by mathematically maximizing the correlation between the weighted average gene importance and evolutionary rate. We further constrain the weighting coefficients to be non-negative because negative coefficients are biologically meaningless. Employing the least squared method in statistics, we can transform this maximization task into a quadratic programming problem. The mathematical representation of the problem is
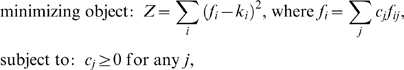
(2)where *k_i_* is the evolutionary rate of gene *i* and *f_i_* is the weighted average importance of gene *i* in all conditions, calculated by averaging gene importance under each condition (*f_ij_*) using non-negative weighting coefficients of the condition (*c_j_*). We solved the quadratic programming problem using the commercial optimization package CPLEX and then calculated the correlation between the weighted average importance of a gene and its evolutionary rate. Note, however, that the above estimation of *c* guarantees the identification of the strongest Pearson's linear correlation between *f_i_* and *k_i_*, but not Spearman's rank correlation. We know of no method that guarantees the identification of the strongest rank correlation between *f_i_* and *k_i_*.

Our results showed that the improvement of the correlation by combining individual conditions is trivial (Table 1). For example, for the 418 experimental conditions, the strongest Pearson's correlation between the weighted average gene importance and *d*_N_ is *r* = −0.2187 (*P*<10^−43^), only 5% stronger than the strongest correlation found among all single conditions (*r* = −0.2082, *P*<10^−39^). Similar results were observed for the other measures of gene evolutionary rate and for combinations of the 10^4^ simulated conditions (Table 1). These results indicate that even weighted average of gene importance across multiple conditions is not strongly correlated with gene evolutionary rate.

**Table 1:** Strongest correlations between gene evolutionary rate and importance measured at different conditions.

Conditions (methods)	Measures of evolutionary rate		
	*d*_N_	*d*_N_ */d*_S_	*PGL*
418 individual lab conditions (experimental)	−0.2082^a^ (1E-39^b^)	−0.1520 (1E-21)	−0.1122 (1E-12)
Combined lab conditions (experimental)	−0.2187 (1E-43)	−0.1580 (1E-23)	−0.1185 (1E-13)
10,000 individual simulated conditions (FBA)	−0.1193 (0.009)	−0.0747 (0.14)	−0.0868 (0.04)
Combined simulated conditions (FBA)	−0.1252 (0.006)	−0.0767 (0.12)	−0.0937 (0.03)
10,000 individual simulated conditions (MOMA)	−0.1354 (0.003)	−0.0748 (0.13)	−0.0941 (0.03)
Combined simulated conditions (MOMA)	−0.1442 (0.002)	−0.0786 (0.12)	−0.1021 (0.02)

a Pearson's correlation coefficient.

b *P*-value.

**Figure 2:**
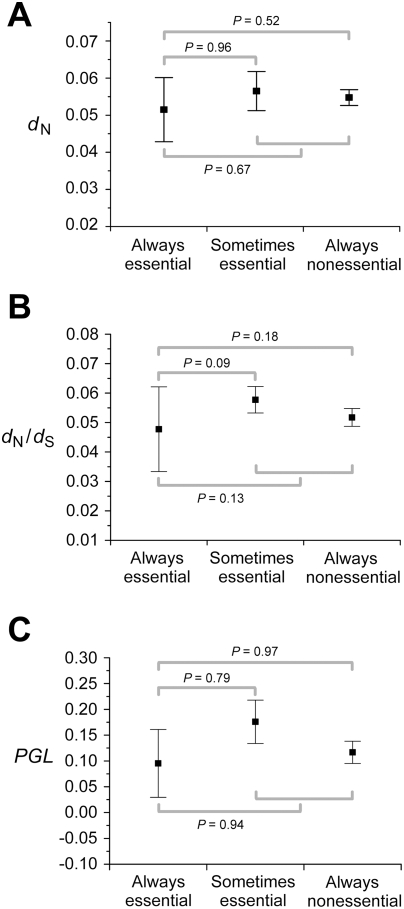
Always-essential enzyme genes do not evolve significantly slower than sometimes-essential and always-nonessential ones, regardless of the measure of the evolutionary rate. Error bars show one standard error. *P*-values are from Mann-Whitney *U* test between groups of genes. The numbers of genes used are 478 for panels A and B and 546 for panel C.

Why doesn't the consideration of so many experimental and simulated conditions and combinations of conditions improve the correlation between gene importance and evolutionary rate? Using FBA, one can classify enzyme genes into three categories according to their importance across multiple conditions: always-essential, sometimes-essential, and always-nonessential. Deleting an always-essential gene causes lethality in all conditions; deleting a sometimes-essential gene causes lethality in some but not all conditions; deleting an always-nonessential gene does not cause lethality in any condition, although it may reduce the fitness of the organism to a non-zero level. Because always-essential genes are as important as or more important than the other two classes of genes in any condition, it is clear that in order to achieve a strong correlation between gene importance and evolutionary rate in any condition or combination of conditions, the evolutionary rate of always-essential genes must be lower than those of the other two classes of genes. Here the enzyme genes are classified into the above three groups based on the essentiality predicted in the 10^4^ simulated conditions. Although the average *d*_N_ of always-essential genes is lower than that of sometimes-essential genes and that of always-nonessential genes, the differences are small and not statistically significant (Figure 2A). The same is true for *d*_N_/*d*_S_ (Figure 2B) and *PGL* (Figure 2C). These results strongly suggest that no single condition or combination of conditions will show a strong correlation between gene importance and evolutionary rate even when more conditions are examined. Thus, if the conditions under which yeast evolved belong to the 418 experimentally examined conditions or are amenable to the current FBA, the lab-nature mismatch hypothesis must be rejected.

### Examining the Correlation between Functional Density and Gene Importance

Equation 1 shows that if functional density (α) and gene importance (β) are independent from each other, evolutionary rate of a gene (*k*) should decrease with the increase of β. The observed weakness of the correlation between gene importance and evolutionary rate prompts us to examine the presumption of independence between α and β, because the correlation between gene importance and evolutionary rate could have been weakened if there is a negative correlation between α and β. By definition, α is the proportion of mutations that destroy the function of a gene, which may be experimentally determined by large-scale site-directed mutagenesis coupled with gene functional assay, a formidable task even for a few genes. In theory, one can use the average number of allowable alternative states across all amino acid sites of a protein to estimate 1−α. But such a measure is currently difficult to acquire at the genomic scale, because it requires the alignments of orthologs from many (i.e., ≫20) divergent species to assure that all potentially allowed amino acids have had chance to appear at any given site. Use of many divergent species greatly increases misidentification of paralogs as orthologs and the risk of comparing functionally-different orthologous proteins, leading to potential overestimation of 1−α. A further complication is that the evolution of a site is often dependent on other sites, meaning that an amino acid is allowed at a site only when another site has a particular amino acid [30],^31^. Thus, the number of allowed amino acids at a site is not a unique number, but rather depends on the genetic background of the same gene or even other genes. Given these difficulties, we decide to use the proportion of amino acid sites within computationally predicted functional domains of a protein to estimate α approximately, because α is expected to be much greater within functional domains than outside domains. This estimation of α is based on the assumption that all sites within functional domains are important to the function of the protein whereas all sites outside domains are unimportant. Although this assumption does not hold in reality, it should not affect our results as long as it does not systematically bias our estimation of α among genes of different β.

Computational algorithms for predicting protein functional domains are based on proteins of known structures and/or amino acid sequences with high evolutionary conservation [32]. There are many available algorithms for protein domain prediction and they are based on different assumptions. Here we employ two widely used methods. The first is the ProSite prediction algorithm [33], which is based on known conserved functional motif sequences. ProSite predictions are relatively conservative and should contain few false positives, as on average only 10% of amino acid sites in a protein are predicted by ProSite to be within functional domains. The second method we used is InterProScan [34], which integrates 13 well known domain prediction algorithms and databases to look for domains. Because InterProScan uses multiple algorithms, its predictions are more comprehensive. To avoid false positive predictions, we consider only those sites that are identified by at least two algorithms of InterProScan as functional domain sites. Under this criterion, on average 47% of protein sites are identified as functional domain sites.

To examine whether the proportion of sites within predicted domains indeed provide information about functional density, we conducted three tests. First, based on the domains predicted by ProSite, we found that sites within domains evolve more slowly than those outside domains in 89% of the yeast genes. The corresponding number is 77% when the domains are predicted by InterProScan. These percentages are significantly greater than the random expectation of 50 percent (*P*<10^−100^, χ^2^ test). Second, the mean *d*_N_ within domains is 40% and 54% that outside domains in ProSite and InterProScan analysis, respectively, both being significantly different from the random expectation of 100% (*P*<10^−50^, paired *t*-test). Finally, we examined if there is a negative correlation between the proportion of sites within domains and the evolutionary rate of the gene, and found the correlation to be ρ = −0.24 (*P*<10^−50^) and −0.56 (*P*<10^−50^), respectively, in ProSite and InterProScan analysis. Taken together, the proportion of sites within predicted domains indeed provide information about functional density and thus may be used as a proxy for α.

**Figure 3:**
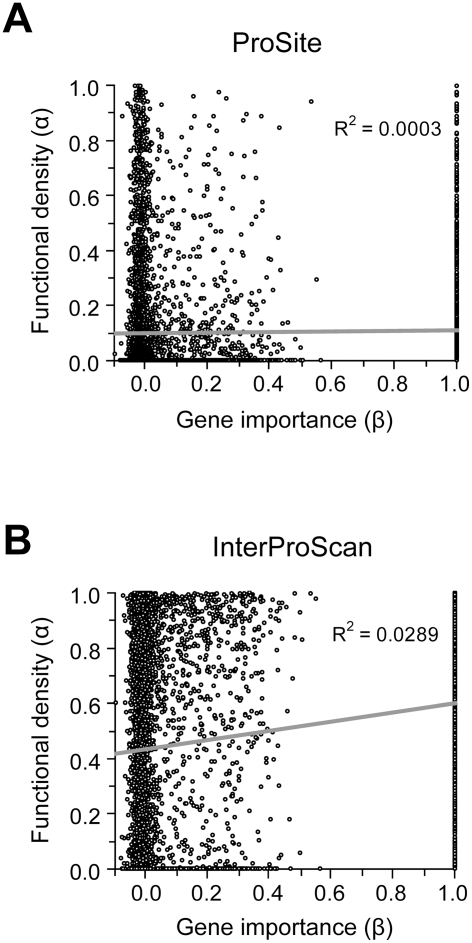
Relationship between the importance (β) and functional density (α) of genes. Gene importance is measured by the experimentally determined fitness reduction upon gene deletion in YPD. Functional density is measured by the proportion of amino acid sites within functional domains predicted by (A) the ProSite algorithm or (B) InterProScan. In InterProScan, a site is considered a domain site when predicted by at least two algorithms. A total of 5936 yeast genes are used in this analysis.

Because our results do not support the lab-nature mismatch hypothesis, we here consider only experimentally measured gene importance under YPD (β). We found very weak positive correlation between α estimated by ProSite and β (ρ = 0.049, *P* = 0.0002) (Figure 3A). If InterProScan predictions are used, there is a stronger positive correlation between α and β (ρ = 0.15, *P*<10^−30^), suggesting that important genes tend to have a higher fraction of functional sites (Figure 3B). We also repeated the analysis under more stringent criteria of InterProScan where a site is considered as a functional domain site only when it is recognized by at least 3 to 6 algorithms. The observed correlation between α and β remains significant (ρ = 0.08–0.12, *P*<0.0001).

However, the above analysis has a confounding factor. Because sequence conservation information is used in predicting functional domains and because important genes tend to be more conserved in sequence (though the correlation is weak), the above observed level of positive correlation between α and β may in part or in total due to the artifact of the analysis. Indeed, we found that after the control of *d*_N_, the partial correlation between α and β becomes ρ = 0.0190 (*P* = 0.240) for the ProSite analysis and ρ = −0.0110 (*P* = 0.497) for InterProScan analysis (≥two algorithms). This result suggests no genuine correlation between α and β. Thus, the weakness of the correlation between gene importance and evolutionary rate is unlikely the result of a potential negative correlation between gene importance and functional density.

### Why Is the Correlation between Gene Importance and Evolutionary Rate So Weak?

Our analysis rejected two frequently proposed explanations of the weakness of the observed correlation between gene importance and evolutionary rate, raising the question of why the correlation is so weak. As mentioned in Introduction, depending on the distribution of the fitness effect of deleterious mutations, the expected correlation may not be strong (Figure S1 and Text S1). In addition, there may be other reasons. Bivariate analysis of yeast data revealed a strong negative correlation between gene expression level and evolutionary rate [35], which led to the recent proposal of the translational robustness hypothesis, asserting that selection against toxicity of misfolded proteins generated by translational errors is the single most important factor governing the rate of protein sequence evolution [36],[37]. This hypothesis explains several factors known to correlate with the rate of protein sequence evolution (e.g., gene expression level and codon usage bias). However, many other rate determinants are known in yeast, including the number of protein interaction partners and gene length, although their impacts on the evolutionary rate are generally much smaller than that of gene expression level [38]. Principal component regression analysis and partial correlation analysis have suggested independent and significant contributions of all these factors [39],[40], although it is not always clear how these factors determine the rate of gene evolution independently from the influence of gene importance [41]. In bacteria and mammals, independent contributions from multiple factors to gene evolutionary rate are also known [4],[5]. Theoretically speaking, the single most important rate determinant is the fraction of mutations that are unacceptable to the gene (α), but this fraction is affected by many biological factors. The fact that the rate of gene evolution is jointly determined by multiple independent factors, some of which are stronger determinants than gene importance, is likely an additional reason why the rate is only weakly correlated with gene importance. To simplify the explanation, let us assume that the rate of gene evolution (*k*) is determined linearly by *n* independent factors (*A*_1_ to *A_n_*) as 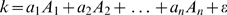, where *ε* represents the statistical error that cannot be explained by the *n* factors and *a*_i_'s are coefficients. Pearson's correlation coefficient between *k* and factor *A_i_* is
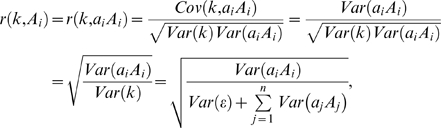
(3)where *Var* stands for variance and *Cov* stands for covariance. Because one rate determinant, gene expression, already accounts for >25% of the variance of *k* [36],[37] and several other factors also make independent and nontrivial contributions [39],[40], the correlation between gene importance and evolutionary rate is much weakened, compared to that when gene importance is the sole contributor.

### Implications for Predicting Functional Importance

**Figure 4:**
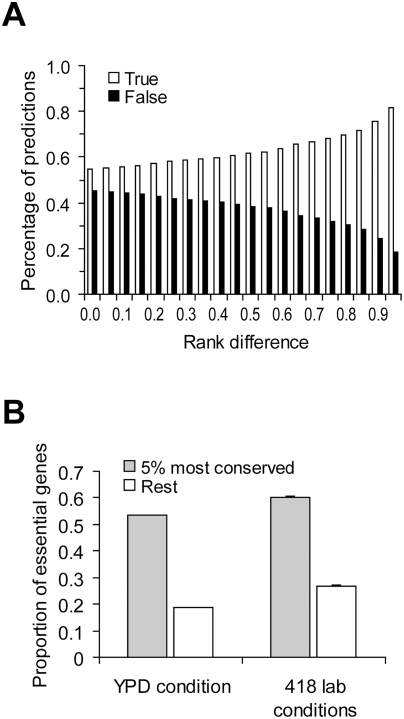
Predictability of the principle of slower evolution of more important genes. (A) Predictions of relative gene importance are more likely to be correct when the difference in evolutionary rate between the two genes under comparison increases. Rank difference shows the minimal fraction of genes in the genome whose ranks in *d*_N_ are between the two genes under comparison. Gene importance is measured by the amount of fitness reduction caused by the deletion of the gene under YPD. For each rank difference criterion, 100,000 random pairs of genes satisfying the criterion are used to estimate the prediction accuracy. (B) Extremely conserved genes (measured by *d*_N_) are more likely to be essential. For the 418 lab conditions, the average proportion of essential genes among the 418 lab conditions and its standard error are shown.

Taken together, we showed empirically that the correlation between gene importance and gene evolutionary rate is weak and showed that this weakness may not be inconsistent with theoretical predictions. In fact, if we randomly pick two yeast genes, the probability that the slower evolving of the two is the more important one is only 54% (based on 100,000 pairs of randomly sampled genes under YPD) (Figure 4A). That is, the prediction based on one of the foremost principles of molecular evolution has a success rate of only 54%, not much greater than that of a pure guess (50%). When the two genes being compared have a larger difference in evolutionary rate, the prediction about their relative importance becomes more accurate, as expected (Figure 4A). For example, we ranked all yeast genes by their evolutionary rates and found that when two genes are separated in rank by over 95% of all genes, the probability that the slower evolving one is more important than the other is 81% (Figure 4A). Essential genes are functionally most important. When the gene importance data from YPD is considered, we found that 55% of the top 5% most conserved genes are essential, whereas only 20% of the remaining 95% of yeast genes are essential (Figure 4B). Similar results are found using the gene importance data from the other 418 lab conditions (Figure 4B). Note that the above demonstrated predictability may not be entirely due to the causal relationship between gene importance and evolutionary rate, because other confounding factors such as gene expression level have not been controlled for. Regardless, our results show that although the correlation between gene importance and evolutionary rate is weak, the principle does have some predictive power when genes of extreme sequence conservation are considered.

### Caveats

There are several caveats in our analysis that warrant discussion. First, experimental measures of gene importance are not without errors. Repeated measures of gene importance under the same conditions showed a correlation as high as 0.92 for the YPD data [23] but a reduced mean correlation of 0.72 for the other 418 lab conditions [19], possibly due to less well controlled experimental procedures in the latter. Thus, the gene importance data we used could potentially explain a maximum of 0.72^2^ = 52% of the variance of the evolutionary rate. But the strongest correlation actually observed was only *r* ^2^ = 4.3% among the 418 individual conditions and 4.8% among combinations of the 418 conditions, both being substantially lower than the theoretical maximum. Similar arguments can be made for the analysis based on computationally predicted gene importance values.

Second, a limitation in using *d*_N_ and *d*_N_/*d*_S_ to measure the rate of gene evolution is that they can be used only for those *S. cerevisiae* genes that have orthologs in the species being compared with (i.e., *S. bayanus*). Our results would not represent a full picture if genes with and without orthologs have drastically different levels of gene importance. To examine this possibility, we compared their importance levels. Because we used reciprocal best hits in BLAST searches to define orthologs, a *S. cerevisiae* gene would not have its operational *S. bayanus* ortholog, if (i) the gene evolved extremely fast, (ii) the gene has been lost in *S. bayanus*, or (iii) the gene has been duplicated in *S. cerevisiae* such that its *S. bayanus* best hit happens to find its paralog to be the best hit. Thus, we separated *S. cerevisiae* genes into singletons and duplicates. We found no significant difference in gene importance between *S. cerevisiae* genes with and without *S. bayanus* orthologs, for either singletons (*P* = 0.11, Mann-Whitney *U* test; Table S1) or duplicates (*P* = 0.63, Table S1). Hence, the potential bias of studying only *S. cerevisiae* genes that have *S. bayanus* orthologs is negligible.

Third, we used three different measures of gene evolutionary rate: *d*_N_, *d*_N_/*d*_S_, and *PGL*. They all have pros and cons, aside from the above consideration. In principle, *d*_N_/*d*_S_ would be the best measure, because it best measures *k*/*u*, which is determined by α and β only, according to Equation 1. Estimates of *d*_N_/*d*_S_, however, suffer from two problems. First, *d*_S_ values may have been saturated because the average *d*_S_ between *S. cerevisiae* and *S. bayanus* is as high as 1.24. Although using more closely related species could improve the estimation of *d*_S_, it would increase the estimation error of *d*_N_ and that of *d*_N_/*d*_S_, due to a reduced number of nonsynonymous substitutions per gene. Second, codon usage bias, prevalent in highly expressed genes of yeast, could lead to underestimation of neutral substitution rates and thus overestimation of *k*/*u*. Because of the positive correlation between the importance of a gene and its expression level [10], codon usage bias causes greater overestimation of *k*/*u* for more important genes, weakening the negative correlation between *k*/*u* and gene importance. If there is little variation in mutation rate among genes, *d*_N_ would be a better index of evolutionary rate for our purpose than *d*_N_/*d*_S_, because estimates of *d*_N_ have smaller sampling errors than those of *d*_N_/*d*_S_. Our results show stronger correlations between gene importance and *d*_N_, compared to that between gene importance and *d*_N_/*d*_S_, suggesting that the disadvantages of using *d*_N_/*d*_S_ outweigh its advantages. Propensity for gene loss (*PGL*) treats each gene as a unit and does not consider the number of substitutions per nucleotide or amino acid site. It is thus conceptually different from the evolutionary rate that Kimura and Ohta [15] and Wilson *et al.* [16] referred to. There are three reasons underlying our observation that gene importance correlates more poorly with *PGL* than with *d*_N_ and *d*_N_/*d*_S_. First, because *PGL* is determined by the fixation of null mutations but not slightly deleterious mutations, it should be less influenced by gene importance, as explained in Introduction and Figure S1. Second, estimation of *PGL* requires genome sequences from a number of species related to the focal species of interest (*S. cerevisiae*). In the present case, *PGL* is estimated from 12 diverse fungi and thus may not accurately reflect the propensity of gene loss in *S. cerevisiae*, because the importance of a gene can change in evolution [10],[24]. Third, estimates of *PGL* potentially have large sampling errors, because the estimated number of losses per gene is quite small.

Fourth, to understand why no single condition or combination of single conditions provides gene importance values that correlate strongly with evolutionary rates, we classified enzyme genes into three groups (always-essential, sometimes-essential, and always-nonessential) and compared their respective evolutionary rates. Due to computational intensity, our classification was based on the FBA analysis of 10^4^ simulated conditions, while in theory it should have been based on all possible conditions. This limitation potentially caused misclassification of some truly sometimes-essential genes as always-essential genes or always-nonessential genes and hence blurred the differences among the three groups. To rectify this problem, we used a strategy that guarantees the identification of all always-essential genes. The metabolic model of yeast allows us to know all nutrients that can be used by this metabolic model. If a gene is essential when all these nutrients are present, it must be essential when one or more of these nutrients are absent. We find that in fact the always-essential genes thus identified are identical to those identified from the 10^4^ simulated conditions. There is, however, no systematic way to guarantee the exact separation of sometimes-essential and always-nonessential genes. We thus merged them and compared this combined group with always-essential genes. Again, we do not find the combined group to have significantly greater *d*_N_, *d*_N_/*d*_S_, or *PGL* than always-essential genes (Figure 2). Thus, our result is true not only for the 10^4^ simulated conditions, but also for all possible combinations of nutrients usable by the yeast metabolic model. Our result differs from that of Papp *et al.* [18] where they found that enzyme genes active in more conditions have lower probabilities of presence in the genomes of 133 diverse species. At least five reasons may account for this difference. First, we counted *PGL* on a known phylogeny of related species using the parsimony method whereas these authors simply calculated the percentage of species that do not have the gene without considering the species phylogeny [18]. Second, most of the species they used are distantly related to yeast and their result is expected to be highly dependent on the choice of species. Third, we considered gene essentiality, a more relevant measure of gene importance than gene activity, because deleting an active gene may or may not have any fitness consequence, depending on alternative pathways in the metabolic network. Fourth, we used a more recent reconstruction of the yeast metabolic network, which is more complete and accurate than the one they used. Fifth and most importantly, because only nine conditions were examined, their result could simply be due to small sample size.

Fifth, Hirsh and Fraser suggested that the correlation between gene importance and evolutionary rate should exist only among genes with relatively low importance [7]. This is because, in Equation 1, *f* quickly declines to virtually 0 when β increases from 0 to 0.1 and any further increase in β has negligible effects on *f* and *k*, although Hirsh and Fraser came to this conclusion using a more complex model [7]. However, we found that the correlation for genes with β<0.1 is extremely weak (ρ = −0.05 for YPD and the strongest ρ = −0.04 among the 418 experimental conditions). We cannot test genes with even smaller β because the accuracy of the estimated β decreases and the number of useable genes decreases. The contradiction between Hirsh and Fraser's prediction and our empirical observation can be understood using Figure S1. Apparently, when there are many slightly and moderately deleterious mutations, use of all genes provides a stronger correlation than using only unimportant genes, because the expected evolutionary rates can still be different between a gene with β = 0.2 and a gene with β = 0.3 (Figure S1K). For example, in Figure S1L, using only genes with β<0.1 gives ρ = −0.36, whereas using all genes gives ρ = −0.83.

Sixth, the correlation between gene importance and evolutionary rate reported here may be in part caused by other co-varying factors. For three reasons, we did not control for confounding factors in our analysis. First, previous authors already determined that the correlation is statistically significant even after the control of confounding factors [3],[10]. Second, our goal here is to discern why the correlation is so weak even when part of it may come from confounding factors. Third, we study the difference in the magnitude of the correlation when various gene importance measures are used; confounding factors such as gene expression level would not affect this difference.

### Conclusions and Implications

Despite the general belief and wide application of the principle that important genes evolve more slowly than less important ones, genomic analysis showed that the correlation between gene importance and evolutionary rate is quite weak. Our analysis does not support the hypothesis that the weakness of the observed correlation is due to the difference between gene importance in the lab and in nature. Furthermore, we found no evidence for the possibility that the correlation is weakened by the potential presence of a smaller fraction of functional sites in more important genes. We conclude that the weakness of the correlation is factual, rather than artifactual. This conclusion is not inconsistent with population genetic predictions, because the predictions vary depending on the prevalence and distribution of the fitness effect of deleterious mutations.

Our result cautions molecular biologists from predicting relative functional importance of genes directly from their relative levels of evolutionary conservation. Nevertheless, our finding that extremely conserved genes are highly likely to be functionally very important may explain the universal perception that the principle of slower evolution of more important genes (or DNA sequences) works well. For example, substantial amount of comparative genomic work aims at using the principle to identify functional non-coding sequences based on their extremely low rates of nucleotide substitution [42]–[45]. An ultra-conserved non-coding sequence is a segment of DNA of over 200 nucleotides with no variation among human, mouse, and rat. Pennacchio et al. found that such ultra-conserved sequences, when they are also conserved between mouse and fish, have a probability of 62% to be actual enhancers during mouse embryonic development [42]. Compared to the virtually zero probability with which a random segment of DNA in the mouse genome is an enhancer, the principle appears to work well. This success is not surprising, because only extremely conserved non-coding sequences are considered. Nevertheless, it should be noted that although a large fraction of extremely conserved non-coding sequences are functional, many functional sequences are not extremely conserved. In other words, the current application of the principle in detecting functional non-coding sequences has a high false-negative rate. Thus far, there has been no evidence that the correlation between sequence importance and evolutionary rate is stronger for non-coding regions than for coding regions. One reason for a potentially stronger correlation for non-coding regions is that several rate determinants in coding sequence evolution simply do not exist in non-coding sequence evolution (e.g., codon usage bias, amount of translation, gene length, and number of protein-interacting partners). In addition, the fraction of mutations that are slightly deleterious may be greater for non-coding regions than for coding regions, given the high modularity of regulatory sequences. In the future when relative importance of many functional non-coding sequences is measured, it will be interesting to examine whether non-coding sequences exhibit a greater correlation between importance and evolutionary rate.

## Materials and Methods

### Yeast Gene Importance Values under YPD and Other 418 Lab Conditions

The fitness values of homozygous-single-gene-deletion yeast strains in the YPD medium [23] were downloaded from http://www-deletion.stanford.edu/YDPM/YDPM_index.html. The corresponding data from the other 418 lab conditions [19] were obtained from http://chemogenomics.stanford.edu:16080/supplements/global/download.html. The microarray raw data were processed by the author-provided Perl scripts and were then normalized to the central mean to yield the relative fitness values of the deletion strains under each condition.

### Yeast Metabolic Network

The metabolic network model of *S. cerevisiae* (iND 750) [25] used in this study was downloaded from the BiGG database (http://bigg.ucsd.edu) and parsed by the COBRA toolbox [46]. The network is composed of 1149 reactions, associated with 750 known genes. Some reactions do not have associated genes because the genes whose protein products catalyze these reactions have yet to be identified. The network model also provides information about stoichiometry, direction of reaction, and gene-reaction association. We followed an established protocol [26] to identify dead-end reactions, which are reactions that must have zero flux under a steady state. These reactions are involved in the generation of metabolites that are neither included in biomass nor transported outside the cell, and may reflect the incompleteness of the metabolic network model. After the removal of dead-end reactions, the yeast metabolic network used in our analysis contains 632 biochemical reactions with 546 associated enzyme genes.

### Flux Balance Analysis (FBA) and Minimization of Metabolic Adjustment (MOMA)

Details of FBA have been described in the literature [21],[22]. Briefly, the flux of each reaction is determined by maximizing the rate of biomass production under the assumption of steady state and the constraints of stoichiometry. We used the optimization package CPLEX (www.ilog.com) to solve the linear programming problem. Gene deletion is modeled by constraining the flux of the corresponding reaction to zero.

MOMA has been previously described in detail [29]. Briefly, MOMA predicts the maximal biomass production rate upon deletion of a reaction by minimizing the differences in all metabolic fluxes between the deletion strain and the wild-type strain. All the constraints used in FBA are still enforced in MOMA. The quadratic programming problem is also solved by CPLEX. As in FBA, deletion of a gene is realized by constraining the flux of the corresponding reaction to zero.

### Simulation of Nutritional Conditions

The natural environments of yeast may change frequently. It is also likely that yeast usually faces nutritionally poor conditions but occasionally encounter rich conditions. To mimic their natural environments, we simulate random nutritional conditions in the following manner. For each condition, we generate a random number *g* from an exponential distribution with a mean of *m* = 0.1 for each of the 103 usable carbon-source nutrients. Here, *g* is the probability that the carbon-source nutrient is available. The actual presence or absence of each nutrient is then determined stochastically using *g*. We then add all required inorganic metabolites. Use of other *m* values (0.05 or 0.5) does not change our results. For each available nutrient, we fix the uptake rate at a random value between 0 and *D* = 20. The actual *D* value used is unimportant and does not alter our result. Only conditions that support the growth of the wild-type cell, as shown by FBA, are considered.

### Separation of Singleton from Duplicate Genes

Singleton and duplicate genes of yeast *S. cerevisiae* are identified by BlastP searches of each gene against all other genes in the genome. A gene is considered as a duplicate if it hits at least one other gene in the genome with the criteria of an E-value = 10^−10^ and an alignable region >50% of the longer sequence. Otherwise, it is treated as a singleton.

### Gene Evolutionary Rates

Following [10], we used the maximum likelihood method to estimate synonymous (*d*_S_) and nonsynonymous (*d*_N_) substitution rates of yeast genes by comparing the orthologous genes of *S. cerevisiae* and *S. bayanus*, which were identified by reciprocal best BLAST hits. The *PGL* information was obtained from a previous study [47], which used the parsimony principle to estimate the number of gene losses on the phylogeny of 12 fungi (*S. cerevisiae*, *S. bayanus*, *S. paradoxus*, *S. mikatae*, *Candida glabrata*, *Kluyveromyces lactis*, *Eremothecium gossypii*, *Debaryomyces hansenii*, *Yarrowia lipolytica*, *Neurospora crassa*, *Kluyveromyces waltii*, and *Schizosaccharomyces pombe*).

### Protein Domain Identification

We downloaded the latest release (Release 20.27) of protein domain scan algorithm ProSite [33] from ftp://ca.expasy.org/databases/prosite/, where an executable program and a compiled domain motif database were available. InterProScan [34] was downloaded from http://www.ebi.ac.uk/Tools/InterProScan/ with the current-release database, and was set up to run locally to identify protein domains.

## Supporting Information

Figure S1 Theoretical expectations of the correlation between gene importance and evolutionary rate under neutral and nearly neutral models. The cumulative probability functions of deleterious effects of random mutations on gene function are shown for the neutral model (A) and the nearly neutral model with three sets of parameters (D, G, J). The expected relationships between *d*_N_/*d*_S_ and gene importance under the four situations are shown in panels B, E, H, K, respectively. When 1000 genes are simulated with measurement errors, the observed relationships between *d*_N_/*d*_S_ and gene importance under the four situations are shown in panels C, F, I, L, respectively, with the blue lines showing the linear regressions. Spearman's rank correlation coefficients and associated *P*-values are shown. The beta distribution that describes the deleterious functional effect of mutations used in panels D, G, and J all have the parameter *b* = 1. The parameter *a* = 10^4^, 10^5^, and 10^6^, respectively, for D, G, and J. Panel M shows Spearman's rank correlation coefficient under different fractions of slightly deleterious mutations. See Text S1 for details.
(0.94 MB PDF)

Figure S2 Frequency distributions of Spearman's rank correlation coefficient ρ between gene importance (i.e., fitness reduction upon gene deletion) and evolutionary rate across 10^5^ simulated nutrient conditions. Gene importance is predicted by FBA. Gene evolutionary rate is measured by (A) nonsynonymous substitution rate *d*_N_, (B) nonsynonymous/synonymous rate ratio *d*_N_/*d*_S_, or (C) propensity for gene loss *PGL*. The yellow arrow in each panel indicates the observed correlation using gene importance values experimentally determined in the YPD medium and the red arrow indicates the strongest correlation across the conditions examined.
(0.36 MB PDF)

Table S1 No significant difference in importance between *S. cerevisiae* genes with and without *S. bayanus* orthologs.
(0.02 MB PDF)

Text S1 Theoretical expectations of the correlation between gene importance and evolutionary rate.
(0.09 MB PDF)
