# Supplementary figures and images for: Why Is the Correlation between Gene Importance and Gene Evolutionary Rate So Weak?
Source: PLoS Genet. 2009 Jan 9;5(1):e1000329. doi: 10.1371/journal.pgen.1000329 (PMC2605560; doi:10.1371/journal.pgen.1000329)

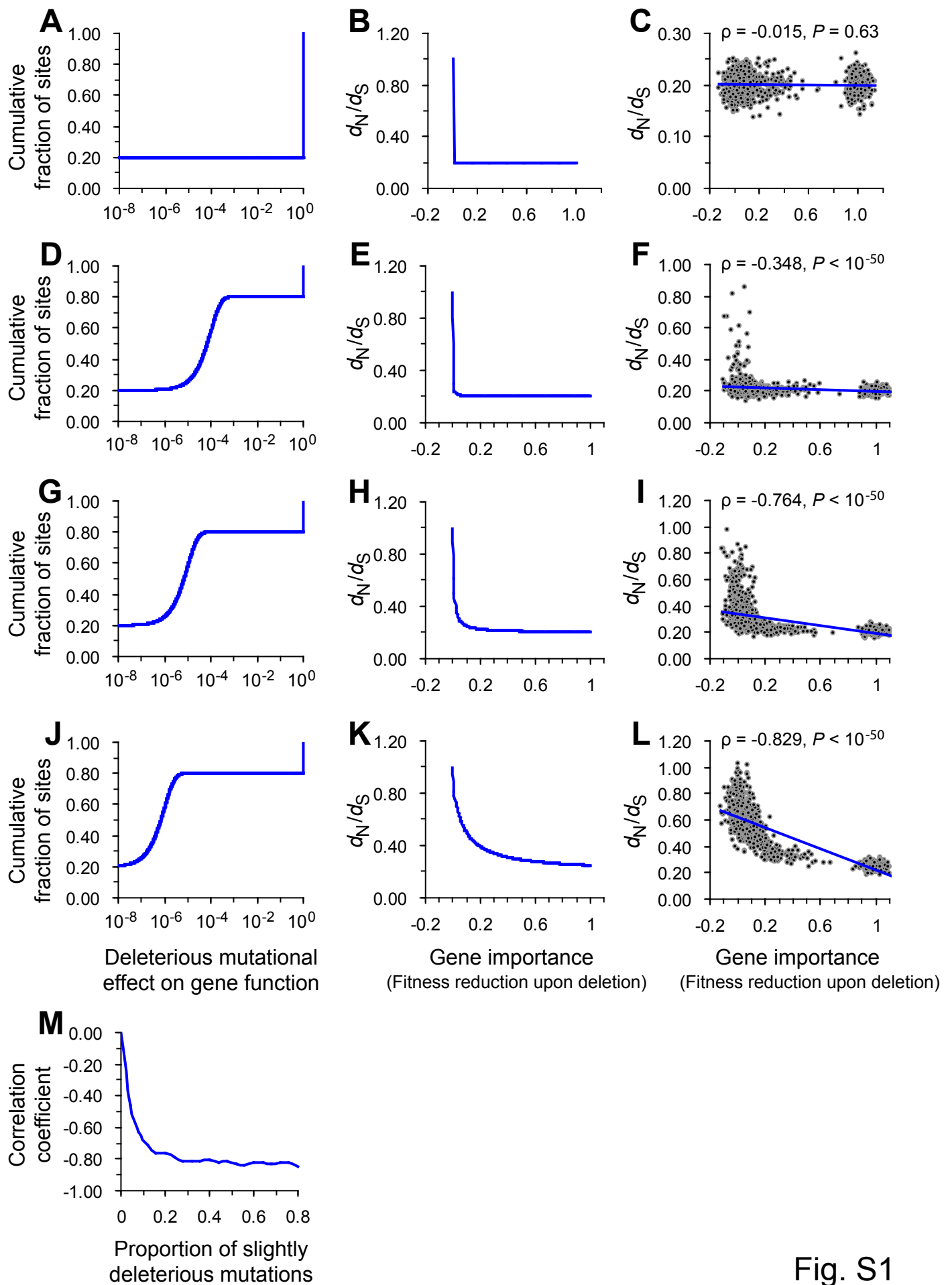

Fig. S1

Supplement: Figure S1 — Theoretical expectations of the correlation between gene importance and evolutionary rate under neutral and nearly neutral models. The cumulative probability functions of deleterious effects of random mutations on gene function are shown for the neutral model (A) and the nearly neutral model with three sets of parameters (D, G, J). The expected relationships between d N/d S and gene importance under the four situations are shown in panels B, E, H, K, respectively. When 1000 genes are simulated with measurement errors, the observed relationships between d N/d S and gene importance under the four situations are shown in panels C, F, I, L, respectively, with the blue lines showing the linear regressions. Spearman's rank correlation coefficients and associated P-values are shown. The beta distribution that describes the deleterious functional effect of mutations used in panels D, G, and J all have the parameter b = 1. The parameter a = 104, 105, and 106, respectively, for D, G, and J. Panel M shows Spearman's rank correlation coefficient under different fractions of slightly deleterious mutations. See Text S1 for details. (0.94 MB PDF) [file pgen.1000329.s001.pdf]

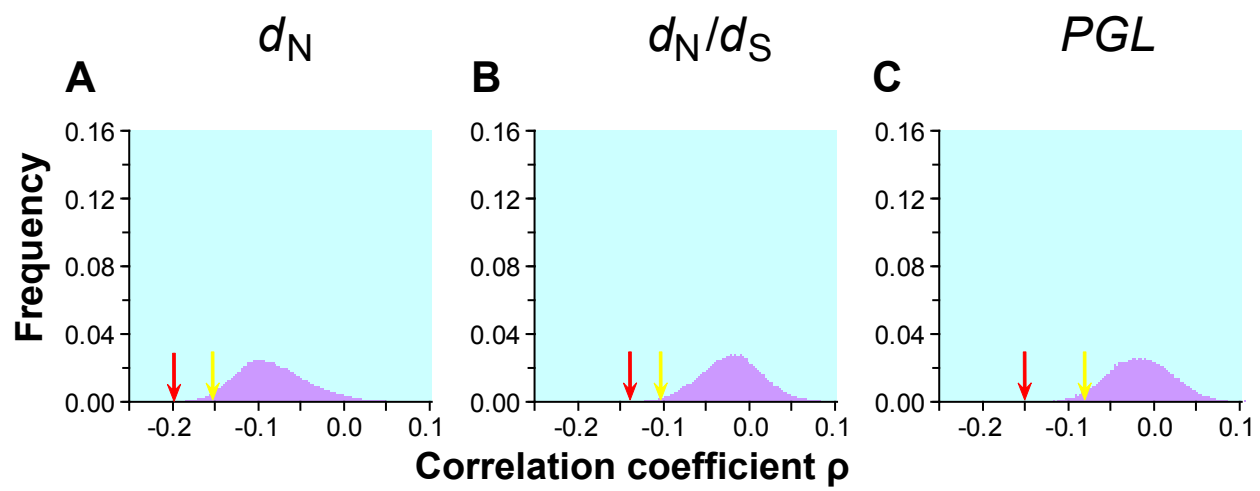

Fig. S2

Supplement: Figure S2 — Frequency distributions of Spearman's rank correlation coefficient ρ between gene importance (i.e., fitness reduction upon gene deletion) and evolutionary rate across 105 simulated nutrient conditions. Gene importance is predicted by FBA. Gene evolutionary rate is measured by (A) nonsynonymous substitution rate d N, (B) nonsynonymous/synonymous rate ratio d N/d S, or (C) propensity for gene loss PGL. The yellow arrow in each panel indicates the observed correlation using gene importance values experimentally determined in the YPD medium and the red arrow indicates the strongest correlation across the conditions examined. (0.36 MB PDF) [file pgen.1000329.s002.pdf]
