# Supplementary material for: Why Is the Correlation between Gene Importance and Gene Evolutionary Rate So Weak?
Source: PLoS Genet. 2009 Jan 9;5(1):e1000329. doi: 10.1371/journal.pgen.1000329 (PMC2605560; doi:10.1371/journal.pgen.1000329)
Supplement: Table S1 — No significant difference in importance between S. cerevisiae genes with and without S. bayanus orthologs. (0.02 MB PDF) [file pgen.1000329.s003.pdf]

Table S1. No significant difference in importance between *S. cerevisiae* genes with and without *S. bayanus* orthologs.

Genes	Mean fitness reduction upon gene deltion in YPD		*P* -values
	With orthologs	Without orthologs	
Singletons	0.288 (2668[a])	0.275 (883)	0.114[b]
Duplicates	0.171 (1331)	0.158 (1104)	0.625

[a] Number of genes in this category

[b] Mann-Whitney U test
