# Supplementary material for: Why Is the Correlation between Gene Importance and Gene Evolutionary Rate So Weak?
Source: PLoS Genet. 2009 Jan 9;5(1):e1000329. doi: 10.1371/journal.pgen.1000329 (PMC2605560; doi:10.1371/journal.pgen.1000329)
Supplement: Text S1 — Theoretical expectations of the correlation between gene importance and evolutionary rate. (0.09 MB PDF) [file pgen.1000329.s004.pdf]

Text S1. Theoretical expectations of the correlation between gene importance and evolutionary rate

In the simplest model of neutral evolution, a mutation is either completely neutral or null. Eq. 1 in the main text describes the relationship between substitution rate and gene importance under this model. Fig. S1A shows the cumulative probability distribution of the deleterious effect of random mutations on gene function, when 80% of mutations are null ($\alpha = 0.8$) and 20% are neutral. This distribution predicts an L-shape curve with a square angle for the relationship between d_N/d_S (i.e., k/u in Eq. 1) and gene importance (Fig. S1B), under the assumption of $N_e = 10^7$ for yeast. We can then estimate the correlation between gene importance and evolutionary rate that can be observed under this model for a large set of genes (e.g., 1000), by considering the distribution of gene importance in yeast, errors in the estimation of gene importance, and errors in the estimation of d_N/d_S. We first classify yeast genes into 10 uniform bins according to their experimentally determined fitness upon deletion (i.e., fitness = 0.0-0.1, 0.1-0.2, 0.2-0.3, …>0.9). We then randomly sample 1000 genes from these bins to represent the genome. For each gene, a uniform random number in the fitness range of the bin to which the gene belongs is assigned to the gene as its true fitness. The true importance value of the gene is one minus the fitness. We assume that the measurement error for gene importance follows a normal distribution with the mean equal to 0 and standard deviation equal to 0.05. To generate an "observed" gene importance value, we randomly generate an error variable following the above distribution and add it to the true gene importance value assigned to the gene. The expected evolutionary rate for every gene can be calculated by Eq. 1 using the true importance value. We assume that the measurement error

of d_N/d_S follows a normal distribution with the mean equal to 0 and standard deviation equal to 10% of the expected value. We similarly generated an "observed" d_N/d_S value for the gene. After generating these values for 1000 genes, we measure Spearman's rank correlation between "observed" d_N/d_S and "observed" gene importance. Our result shows that a significant correlation between "observed" gene importance and "observed" evolutionary rate is not expected under this simple neutral model (Fig. S1C).

However, the situation could be different when there is a large fraction of mutations that only slightly or moderately impair the function of a gene [2,3]. Let the selection coefficient against a slightly/moderately deleterious mutation be $e\beta$, where e is the deleterious effect of the mutation on gene function and β is the importance of the gene as defined in the main text. Following a previous study [4], we assume that e follows a beta distribution. A beta distribution has two parameters, a and b. The mean of the distribution is $a/(a+b)$ and the variance is $ab/[(a+b)^2(a+b+1)]$. We here examine three sets of parameters for the beta distribution because the mean functional effect of slightly/moderately deleterious mutations in real genes is unknown. As in the previous section, we still assume that 20% of the mutations are completely neutral. We further assume that 20% of the mutations are null. The remaining 60% of the mutations follow the beta distribution (Fig. S1D, G, and J). As the functional effects of most non-null deleterious mutations get smaller, the L-shape curve for the relationship between d_N/d_S and gene importance starts to have a round angle. Following the same simulation strategy described above, we sample 1000 genes under this model for each parameter set. We found that for all three parameter sets examined, the correlation between gene importance and evolutionary rate is statistically significant (Fig. 1F, I, and L).

In particular, when the beta distribution has the parameters of $a = 10^6$ and $b = 1$, this correlation can reach ρ = -0.83, suggesting that a strong correlation is theoretically possible under a more realistic model of evolution. Note that under the above parameter set, the deleterious functional effects of non-neutral, non-null mutations are concentrated between 10^{-7} and 10^{-5} (Fig. 1J). Because the mean gene importance is 0.3 in yeast, these mutations have a deleterious fitness effect between $N_e s = 0.3$ and 30 for an average gene, where s is the selection coefficient against the mutation. This range of $N_e s$ fits the classic definition of slightly to moderately deleterious mutations [2]. The model with the other two parameter sets has little slightly deleterious mutations but much more moderately deleterious mutations.

Because the proportion (x) of slightly/moderately deleterious mutations in yeast genes is unknown, we further examine the effect of x on the correlation between gene importance and evolutionary rate. To achieve this goal, we still assume that 20% of the mutations are neutral, but x varies between 0 and 0.8, whereas the proportion of null mutations equals $0.8\text{-}x$. Similar to the above analysis, we simulate 1,000 genes and calculate their "observed" gene importance and "observed" evolutionary rate with different fractions of slightly/moderately deleterious mutations. Here, the parameter set of $a = 10^6$ and $b = 1$ is used for the beta distribution because this parameter set best reflects slightly and moderately deleterious mutations for yeast, as aforementioned. Our result shows that as long as there are at least 5-10% mutations that are slightly/moderately deleterious, the correlation between gene importance and evolutionary rate is substantial (Fig. S1M).

References

1. Kimura M (1983) The Neutral Theory of Molecular Evolution. Cambridge: Cambridge University Press.
2. Ohta T (1992) The nearly neutral theory of molecular evolution. Ann Rev Ecol Evol 23: 263-286.
3. Ohta T (1973) Slightly deleterious mutant substitutions in evolution. Nature 246: 96-98.
4. Hirsh AE, Fraser HB (2001) Protein dispensability and rate of evolution. Nature 411: 1046-1049.
